# Accumulation of Bioactive Lipid Species in LPS-Induced Neuroinflammation Models Analysed with Multi-Modal Mass Spectrometry Imaging

**DOI:** 10.3390/ijms252212032

**Published:** 2024-11-08

**Authors:** Irma Berrueta Razo, Kerry Shea, Tiffany-Jayne Allen, Hervé Boutin, Adam McMahon, Nicholas Lockyer, Philippa J. Hart

**Affiliations:** 1 Medicines Discovery Catapult, 35 Mereside Alderley Park, Macclesfield SK10 4ZF, UK; kerry.shea@md.catapult.org.uk (K.S.);; 2 The Photon Science Institute, Department of Chemistry, The University of Manchester, Manchester M13 9PL, UK; nick.lockyer@manchester.ac.uk; 3 Division of Imaging, Informatics and Data Sciences, Faculty of Biology, Medicine and Health, University of Manchester, Manchester M13 9PL, UK; herve.boutin@inserm.fr (H.B.); adam.mcmahon@manchester.ac.uk (A.M.); 4 Geoffrey Jefferson Brain Research Centre, Northern Care Alliance, Manchester Academic Health Science Centre, University of Manchester, Manchester M13 9PL, UK; 5 INSERM, U1253 iBrain, Université de Tours, 37020 Tours, France

**Keywords:** mass spectrometry imaging, DESI, MALDI, TOF-SIMS, neuroinflammation

## Abstract

Neuroinflammation is a complex biological process related to a variety of pathologies, often requiring better understanding in order to develop new, targeted therapeutic interventions. Within this context, multimodal Mass Spectrometry Imaging (MSI) has been used to characterise molecular changes in neuroinflammation for biomarker discovery not possible to other techniques. In this study, molecules including bioactive lipids were detected across inflamed regions of the brain in rats treated with lipopolysaccharide (LPS). The detected lipids may be acting as inflammatory mediators of the immune response. We identified that N-acyl-phosphatidylethanolamine (NAPE) species accumulated in the inflamed area. The presence of these lipids could be related to the endocannabinoid (eCB) signalling system, mediating an anti-inflammatory response from microglial cells at the site of injury to balance pro-inflammation and support neuronal protection. In addition, polyunsaturated fatty acids (PUFAs), specifically n-3 and n-6 species, were observed to accumulate in the area where LPS was injected. PUFAs are directly linked to anti-inflammatory mediators resolving inflammation. Finally, acylcarnitine species accumulated around the inflammation region. Accumulation of these molecules could be due to a deficient β-oxidation cycle.

## 1. Introduction

Tissue injury causes an inflammatory response that promotes wound healing and fights infection [1,2]. Neuroinflammation is a critical event in brain injury and in the development of neurodegenerative diseases like Alzheimer’s disease (AD), Parkinson’s disease (PD), multiple sclerosis (MS) and dementia [3,4,5,6]. Understanding all biological aspects of neuroinflammation, such as the role played by innate immune cells during this process and the inflammatory mediators involved, is crucial for the development of targeted therapeutics for these pathologies [1,2].

Mass Spectrometry Imaging (MSI) is a powerful technique used to visualise the spatial distribution of small molecules, that are not easily accessible to other, more classical approaches and without a priori knowledge, hence enabling access to complex chemical information from biological specimens including brains. With MSI, it is possible to identify molecular changes induced by processes including neuroinflammation [7,8,9,10,11,12,13]. Imaging techniques such as Matrix-Assisted Laser Desorption Ionisation (MALDI) have been applied to the analysis of cerebral inflammation derived from focal cerebral ischaemia in mouse brain models [14]. In a study by Nielsen et al., bis(monoacylglycero)phosphate (BMP) lipid species were identified in the ischaemic area. The group also used Desorption Electrospray Ionisation (DESI) mass spectrometry imaging for the detection of N-acyl-phosphatidylethanolamine (NAPE) lipid species accumulating at the injury site during the ischaemic event. Both groups of phospholipids, BMPs and NAPEs, are not commonly detected in healthy brain samples. For this reason, the authors hypothesised that BMPs and NAPEs could be biomarkers of phagocytising macrophages/microglia and dying neurons, respectively [14]. In a separate study, brain sections from an ischaemia rat model were analysed with DESI-MSI to investigate the spatial distribution of NAPE lipid expression following an ischaemic event. Different NAPE lipid species were observed accumulating in the injury area after 24 and 48 h of the ischaemic event [15]. In a different study, non-localised neuroinflammation was induced by administering LPS intraperitoneally in mouse models and a change in lipid dynamics was identified using DESI imaging. In this study, behavioural assessments indicated a neuroinflammation effect from the LPS treatment, where lipids including phosphatidylethanolamine (PE) and docosatetraenoic acid were proposed as potential biomarkers of neuroinflammation. Additionally, the levels of activated microglia were elevated in the hippocampus region [16].

Hankin et al., in a publication focusing on MALDI mass spectrometry imaging of rat models of traumatic injury and ischaemia, reported an increased presence of a ceramide species related to apoptosis. An alteration in the concentration of Na^+^ and K^+^ phospholipid adducts was also found [17]. A more recent study applied Time-of-flight Secondary Ion Mass Spectrometry (TOF-SIMS) to image traumatic injury rat models to identify changes in the brain lipidome at high spatial resolution [18]. In this study the authors focused on the depletion of structural cardiolipin species at the injury site.

Furthermore, a report from 2018 described the 3D detection of long-chain acylcarnitines in spinal cord injury models using MALDI imaging [19]. The acylcarnitines appeared to be heterogeneously distributed around the margins of the spinal cord lesion. The identification of long-chain acylcarnitine species was co-localised with the presence of resident macrophages/microglia that were detected with immunofluorescence [19]. However, the action of acylcarnitines and their link to neuroinflammation has not been widely explored.

## 2. Results and Discussion

**Figure 1:**
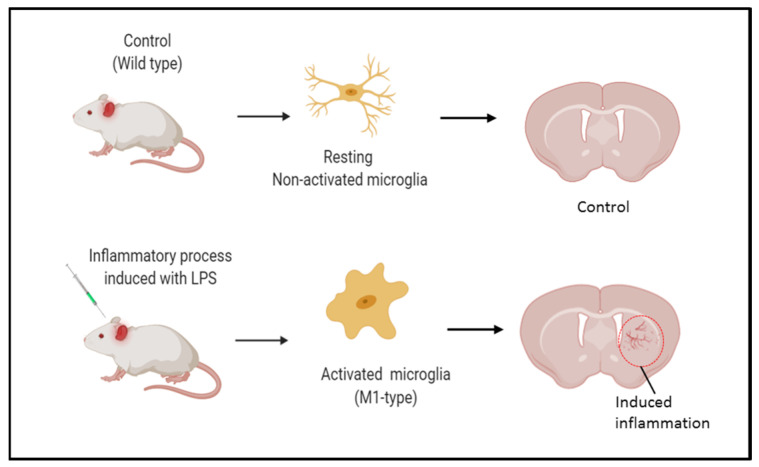
Preclinical models of neuroinflammation. Lipopolysaccharide from *E. coli* is injected in the brain to induce an inflammatory response. LPS increases the expression levels of proteins, promoting the activation of microglia [3].

In the present study, we developed an untargeted multi-modal imaging approach including MSI techniques such as MALDI, DESI, and TOF-SIMS to assess changes in the brain lipidome, in a rat model of bacterial -induced neuroinflammation that was confirmed by immunohistochemistry and microscopy. This LPS model of neuroinflammation is a well-stablished methodology that promotes an inflammatory response in the brain inducing the activation of microglia and production of cytokines (Figure 1) [3].

For this exploratory project we had access to different analytical imaging techniques that were applied to the untargeted analysis of serial sections from the same fresh-frozen brain models. This multi-modal approach was implemented to find complementary information about the lipid species that are present during neuroinflammation, to overcome the limitations of individual techniques such as spatial resolution and sample-throughput, and to validate the results obtained with different imaging modalities. To our knowledge, there is not a single technique that will capture all the biological aspects of complex biological processes, like neuroinflammation. Each MSI technique applied here offers distinct capabilities and limitations due to their distinctive ionisation methods and feasible pixel size [7,8,9,10,11,12,13]. For instance, DESI uses an electrospray to ionise the surface of a sample under ambient conditions with limited spatial resolution (100 µm/pixel in our experiments). In this study, DESI was applied to the analysis of lipid species from complete brain sections. On the other hand, MALDI uses a laser beam to ionise biomolecules in vacuum from the surface of a sample covered in crystallised matrix. In our experiments, we used 2,5-Dihydroxybenzoic acid as the matrix to ionise lipid species across the brain sections with a pixel size of 50 µm. This approach allowed us to detect different lipids species to those observed with DESI due to the difference in ionisation method. Finally, TOF-SIMS relies on highly focused ion beams for sample ionisation in high vacuum. Analysis of preclinical brain samples with giant cluster ion beams by TOF-SIMS has proven particularly successful for higher spatial resolution lipid imaging of small regions of tissues [18]. In this study, we applied an ion beam with 25,000 water molecules with 5 µm of diameter to characterise intact lipid molecules from our neuroinflammation models. With this spatial resolution, it takes a long time to do sample analysis and therefore this technique is better suited to analysing small regions of interest. For this exploratory study, we selected small regions where LPS was injected to see the molecular distribution in more detail. Additionally, we also obtained microscopy images to evaluate the histological structure and presence of activated microglia with histological staining. This approach was applied to localise the regions in the sample with activated microglia. Images from all the modalities mentioned above enabled the acquisition of complimentary information from lipid species present during the development of neuroinflammation in our preclinical models.

**Figure 2:**
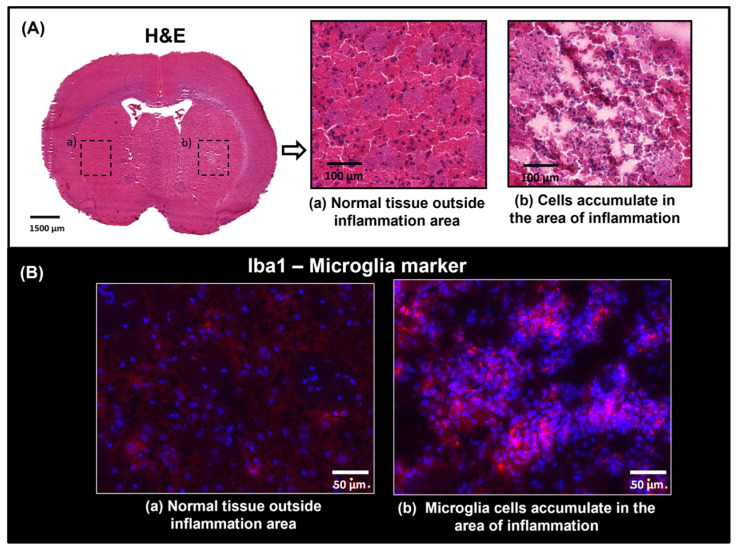
(**A**) H&E staining on a section treated with LPS. Optical images from the whole section and area of inflammation are shown. An accumulation of cells stained with Haematoxylin (blue) is observed in the area of inflammation (b), indicating possible infiltration of immune cells during the neuroinflammatory process. A region without inflammation from a corresponding region in the opposite hemisphere is also included (a), showing normal histomorphology in the striatum. (**B**) Immunofluorescence staining in consecutive section, showing the area where anti-Iba1 (in red) binds to resting or activated microglia.

Sections from LPS-treated and non-treated brains (Figure 2) were stained with Haematoxylin and Eosin to assess tissue morphology and cellular accumulation in the inflammation area. The same areas from serial sections were stained with the antibody anti-Iba1 which targets the protein Iba1, used as a marker of microglia/macrophage activation in immunohistochemistry. Immunofluorescence staining was used to to identify the presence of microglia with anti-Iba1 (red-fluorescent dye, Figure 2) and DAPI was used as a nuclear counterstain (blue-fluorescent dye, Figure 2). During histological processing, we observed impaired tissue integrity where neuroinflammation was induced with LPS. This made immunostaining challenging, resulting in some tissue loss during the multiple tissue washes required in this process. Microscopy images indicate an accumulation of cell bodies in the neuroinflammation region (Figure 2A), suggesting the proliferation and infiltration of cells to this area. Immunofluorescence staining showed the presence and activation of microglia at the site of injury (Figure 2B). This was the case for all the brains treated with LPS, either harvested after 24 or 48 h. These findings were more evident when comparing the region of inflammation with an area without inflammation. Serial sections from the same brains were characterised with MSI, including imaging of whole brain sections with MALDI and DESI and analysis of smaller regions using TOF-SIMS with higher spatial resolution.

### 2.1. Accumulation of N-Acyl-Phosphatidylethanolamine Lipids as a Response of Neuroinflammation

DESI-MSI reveals a number of new signals in the LPS-treated samples which we have putatively assigned NAPE (N-acyl-phosphatidylethanolamine) lipid species (Supplementary Table S1). NAPEs are glycerophospholipids composed of PE and three fatty acid chains, one of them linked to the PE head group via an amide bond [20,21]. Each NAPE species has variations in length and number of double bonds in each fatty acid chain. NAPE lipids are minor structural components in mammalian membranes, representing 0.01% of the total phospholipid content [21]. NAPE lipids are precursors of N-acylethanolamines (NAEs) by the action of a phospholipase D, through the endocannabinoid signalling pathway. NAEs are bioactive lipid mediators that regulate a series of physiological functions, including inflammatory processes, neuroprotection and cell proliferation [21].

**Figure 3:**
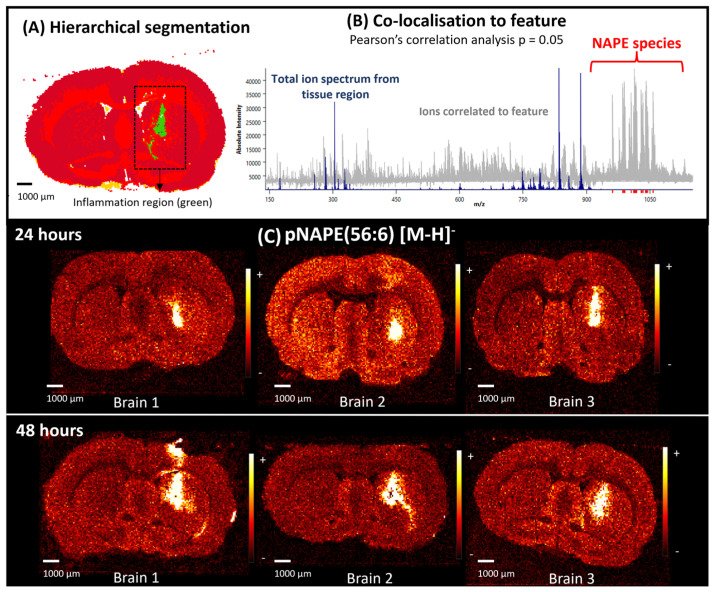
(**A**) Segmented total ion image by hierarchical clustering from a brain treated with LPS. The segmentation allowed the identification of regions that have spectral differences. Here, the inflammation region (green) was identified and selected for further analysis. (**B**) Co-localised values to a feature. Pearson correlation analysis was applied to obtain the ions specifically co-localised to the inflammation regions of brains treated with LPS. This was calculated using SCILS Lab with *p* = 0.05. Total ion spectra from the tissue region are shown in blue and the values correlated to the inflammation region are displayed in grey. NAPE lipid species were some of the ions co-localised to the inflammation area. (**C**) Single ion images of pNAPE(56:6) from all the biological replicates in the two time points (24 and 48 h). Accumulation of the NAPE lipid species was observed in the inflammation area in all the LPS-treated brains. All images were normalised to the total ion count.

In our LPS-treated samples, we were able to detect an accumulation of a plasmalogen-type NAPE, pNAPE(56:6) at *m*/*z* 1012.80, NAPE(56:6) at *m*/*z* 1028.79 and NAPE(56:0) at *m*/*z* 1040.85 using DESI imaging in negative ion mode. Accumulation of these lipids species was observed across the inflammation regions of all biological replicates treated with LPS after 24 and 48 h (Figure 3, Supplementary Figure S1). The signal intensity of the NAPE ions *m*/*z* 1012.80, *m*/*z* 1028.79 and *m*/*z* 1040.85 was sufficient to perform MS/MS experiments to corroborate the ion assignment (Supplementary Information). To process the data, images from the LPS-treated samples were segmented using hierarchical clustering to separate the different regions of the images based on their chemistry. From the segmented images, the inflammation regions were identified. Pearson correlation analysis was applied to elucidate the ions co-localised to the identified inflammation regions. Only statistically significant correlations (*p* = 0.05) were selected. Figure 3 shows one of the segmented images with its total ion spectrum as well as the co-localised values. The co-localised values between *m*/*z* 900 and 1100 are included in the supplementary information (Table S2). Some of these ions were putatively assigned as NAPE lipid species.

**Figure 4:**
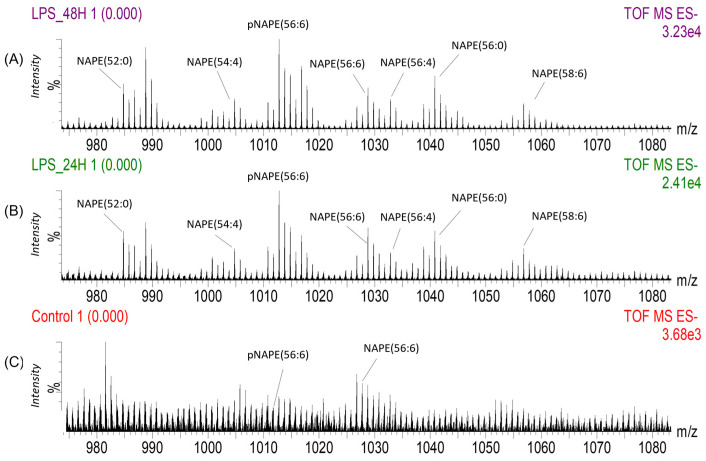
Summed total ion DESI mass spectra extracted from regions of interest with 100 pixels from (**A**) The inflammation area of the brain striatum treated with LPS and harvested after 48 h, (**B**) The inflammation region the striatum harvested after 24 h and (**C**) Region of the striatum from a non-treated brain (Control). These spectra show the higher signal intensity detected from several NAPE lipid species in the LPS-treated brains. Two of these NAPE species, pNAPE(56:6) and NAPE(56:6) are also detected in the control brain but their signal intensity is lower compared to the brains with inflammation, and they do not accumulate in any areas of the striatum.

The intensity of the signal detected from NAPE species was higher in the inflammation region, when compared to a similar area from a control brain (Figure 4). To achieve this comparison between images, all data sets were processed and normalised together to the total ion count. Within this mass range, we also observed species including NAPE(52:0) at *m*/*z* 984.77, NAPE(54:4) at *m*/*z* 1004.80, NAPE(56:4) at *m*/*z* 1032.81 and NAPE(58:6) at *m*/*z* 1056.84. These ions were assigned according to their exact masses [22] as their signal intensity was not sufficient to perform fragmentation by MS/MS (Supplementary Table S1, Figures S2–S4). We did not detect any accumulation of these ions we have assigned to NAPEs in control brains (Figure 4).

**Figure 5:**
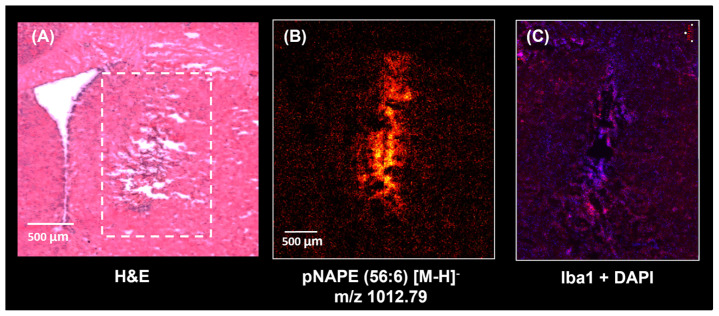
(**A**) Microscopy image showing a H&E staining with the inflammation region highlighted by the white dotted line box. (**B**) Accumulation of pNAPE(56:6) detected with TOF-SIMS in the neuroinflammation region. This image was acquired on a consecutive section in negative ion mode with 5 µm of spatial resolution. (**C**) Histological assessment on a serial section from the same brain also revealed microglial accumulation observed with Immunostaining, showing cell nuclei stained with DAPI in blue and microglia stained with Iba1 in red.

NAPE species were also observed when imaging serial sections using TOF-SIMS at a higher spatial resolution, as shown in Figure 5 and Figure S5 (Supplementary Information). Figure 5 displays a TOF-SIMS image focused on the neuroinflammation region, histological staining with H&E, and immunofluorescence. This image confirms the up-regulation of pNAPE(56:6) in the injury site and accumulation of cells and microglia. We did not observe accumulation of microglia outside the inflammation region (Figure 2). TOF-SIMS spectra from regions with and without inflammation were extracted and compared (Supplementary Figure S5). The main chemical differences between regions can be observed between *m*/*z* 900 and 1100, where different NAPE lipid species were putatively identified in the area with inflammation.

The accumulation of NAPE lipids in the inflammation site and its correlation to the presence of microglia in the same region could be explained through the endocannabinoid (eCB) signalling system [23,24,25,26,27]. The eCB system is activated in response to the neuroinflammatory event promoted by the LPS injection [23,27]. In the eCB system, NAPE lipids are converted to the neurotransmitter anandamide that targets the cannabinoid receptors (CB) [28]. During neuroinflammation, microglia express functional eCBs that target the CB receptors to mediate the local immune response [27]. During LPS-induced neuroinflammation, the microglia cells observed at the injury site could be playing an anti-inflammatory role to protect the neurons and help maintain the brain homeostasis [21,23,29,30].

### 2.2. Detection of Polyunsaturated Fatty Acids (PUFAs) in Neuroinflammation Regions

**Figure 6:**
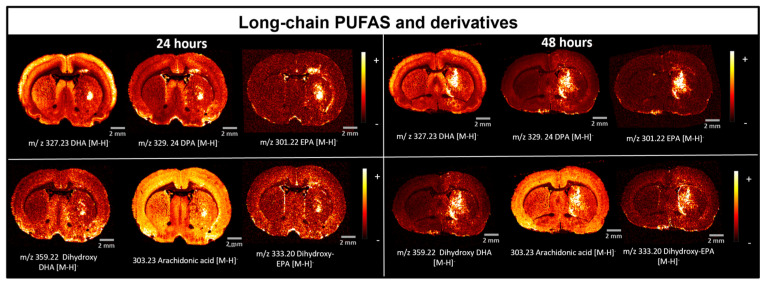
Accumulation of long-chain PUFAs and their derivatives in the inflammation region detected by DESI-MSI. The PUFA species n-6 and n-3, as well as their possible hydroxy (Supplementary Data) and dihydroxy derivatives were detected in the inflammation region. The n-3 species detected are EPA, DPA and DHA. AA is also detected, and it is part of the n-6 fatty acid family.

High levels of polyunsaturated fatty acid species (PUFAs) were detected in brains injected with LPS after 24 and 48 h, in particular, n-3 PUFA species and n-6 arachidonic acid (AA). The n-3 lipids detected are docosahexaenoic acid (DHA) at *m*/*z* 327.23, docosapentaenoic acid (DPA) at *m*/*z* 329.24 and eicosapentaenoic acid (EPA) at *m*/*z* 301.22. AA was observed at *m*/*z* 303.23. All PUFAs were detected as [M-H]^−^ species and were assigned based on their exact mass (Figure 6 and Supplementary Table S3).

Similar to NAPEs, n-3 PUFAs and AA were mainly present within the inflammation site (Figure 6). The role of PUFAs in neuroinflammation has been studied extensively, especially in the landscape of inflammation. PUFAs influence the inflammatory response by mediating a series of mechanisms or by changing the fatty acid composition of cell membranes [31,32,33,34,35,36]. These structural changes affect cell signalling and production of PUFA-derived lipid mediators [37]. AA is a structural lipid found in cell membranes as its esterified covalently bound form. During inflammation AA is released and oxygenated, acting as a precursor of eicosanoid molecules although eicosanoid molecules can also be derived from n-6 PUFAs including DHA, DPA and EPA [35]. The eicosanoid family are a group of bioactive specialised lipid mediators that regulate inflammation and cytokine production [38]. Eicosanoids have different inflammatory properties depending on the fatty acid they are derived from. For example, if they are derived from n-6 fatty acids such as EPA or DHA, they have anti-inflammatory properties whereas eicosanoids derived from n-6 PUFAs like AA have pro-inflammatory functions [31,35,36,38].

EPA, DPA and DHA are precursors of specialised anti-inflammatory mediators including resolvins [35]. Hydroxy and dihydroxy species derived from these long-chain PUFAs were detected in our neuroinflammation models and were assigned according to their exact masses (Supplementary Table S3). These fatty acid-derivative molecules co-localise with the presence of n-6 and n-3 PUFAs and with microglia cells observed by immunofluorescence (Figure 2 and Figure 6). This supports their biological roles as inflammatory mediators as precursors of anti-inflammatory mediators. Although these molecules are directly linked to the immune response, previous work has also suggested that fatty acid levels could also be upregulated as a result of hypoxia post-mortem [14,39].

### 2.3. Accumulation of Acylcarnitines as a Result of Incomplete β-Oxidation

**Figure 7:**
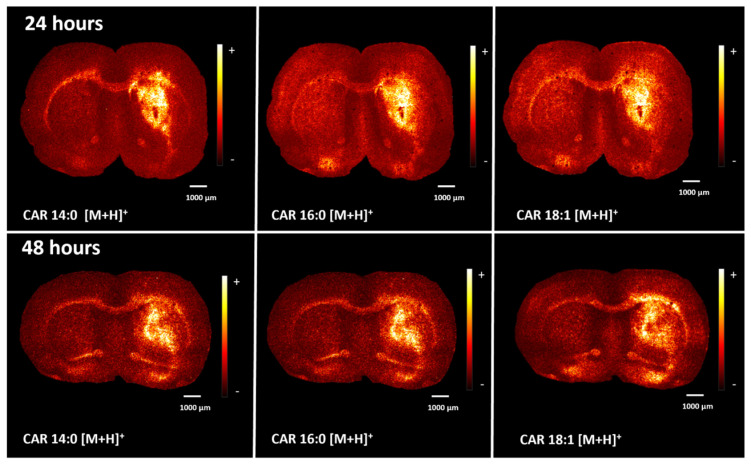
Accumulation of Fatty acylcarnitines surrounding the inflammation region detected with MALDI-MS after 24 and 48 h of the LPS injection. Single ion images showing the detection of tetradecanoylcarnitine at *m*/*z* 372.31, palmitoylcarnitine at *m*/*z* 400.34, CAR 18:1 at *m*/*z* 426.35 and stearoylcarnitine at *m*/*z* 428.37. These fatty acyl carnitine species seem to be upregulated in the right hemisphere of the brain where the inflammation event was induced and are detected in higher intensity around the area of inflammation.

Accumulation of acylcarnitines was observed in the LPS-induced neuroinflammation models with MALDI and DESI imaging in positive ion mode harvested 24 and 48 h after the LPS injection. Figure 7 includes ion images showing the distribution of acylcarnitine species detected with MALDI. The species observed are tetradecanoylcarnitine (CAR 14:0) at *m*/*z* 372.31, palmitoylcarnitine (CAR 16:0) at *m*/*z* 400.34, CAR 18:1 that could be assigned as elaidic carnitine or o-oleoylcarnitine at *m*/*z* 426.35 and stearoylcarnitine (CAR 18:0) at *m*/*z* 428.37. These species were assigned according to their exact masses (Supplementary Table S4) as we could not detect enough signal to perform MS/MS in situ. The acylcarnitine ions are found in the same hemisphere of the brain where neuroinflammation developed, surrounding the injury region. These ion species were detected with DESI imaging in positive ion mode (Figure 7 and Supplementary Figure S7). Accumulation of acylcarnitines was not observed when analysing samples from control brains. The abnormal accumulation of acylcarnitine molecules surrounding the area of inflammation could indicate an increase in local oxygen demand leading to a hypoxic-driven β-oxidation cycle to break down fatty acids [29,40]. Alternatively, the accumulation could be linked to tissue oxidative damage and the subsequent dysfunction of the fatty acid β-oxidation cycle. This results from mitochondrial malfunction due to the abnormal production of reactive oxygen species (ROS) derived from proinflammatory molecules during acute neuroinflammation, such as LPS-induced neuroinflammation [41,42,43].

Acylcarnitines are molecules that contain a carnitine molecule conjugated to a fatty acid chain [44]. They play an essential role during the oxidation of long-chain fatty acids to produce ATP. During this process, acylcarnitines are transported inside the mitochondria by the action of carnitine acylcarnitine translocase (CACT). CACT shuttles the acylcarnitine molecules through the mitochondrial membrane. Once inside the mitochondria, the carnitine part of the molecule is detached and returned to the cytosol to repeat the cycle while the Acyl-CoA part of the molecule is oxidised. The result is to generate ATP and contribute to regulated cellular energy production [44,45]. Cellular accumulation of acylcarnitines could implicate a disruption in the transport system and indicates oxidative stress [29,46]. A number of studies have hypothesised that long-chain acylcarnitines can accumulate during inflammation as a consequence of incomplete β-oxidation of fatty acids [44,45]. A previous study carried out on mouse macrophages suggested that acylcarnitines, the by-products of deficient β-oxidation, activate downstream pro-inflammatory signalling pathways [46,47,48]. In our LPS models, the accumulated acylcarnitines around the inflammation could, on this basis, be mediating a pro-inflammatory response from the host, although the details of this mechanism are not fully known and the specific molecules involved are yet to be identified.

## 3. Materials and Methods

This project focused on the lipidomic changes during progression of neuroinflammation using multi-model imaging. We investigated two timepoints; 24 h and 48 h in a preclinical model of centrally administered LPS-induced neuroinflammation which triggers microglia activation and expression of pro-inflammatory cytokines.

### 3.1. Animal Models

Eight adult male Wistar rats were used in this study. All Animal experiments were conducted in accordance with the U.K. Animals (Scientific Procedures) Act 1986 and had institutional and regulatory ethical approval (University of Manchester Animal Welfare and Ethical Review- AWERB) under Home Office license in accordance with ARRIVE guidelines. All the methods are reported according to the ARRIVE guidelines. The animals were kept under a 12-h light-dark cycle with free access to food and water. For all procedures, animals were anaesthetised by isoflurane (5% induction, maintenance 2%) inhalation in O_2_/NO_2_. The 6 rats in total were divided into two groups of 3. They all received an intrastriatal stereotactic injection (Bregma +0.7 mm, lateral −3.0 mm, depth 5.5 mm from the surface of the brain) of 10 µg of LPS in saline (from *Escherichia coli* 055:B5, Sigma Aldrich, Poole, UK). The first group was euthanised 24 h post-injection whereas the second group was euthanised 48 h post-injection. Two extra animals were kept untreated to be used as controls. All LPS-treated and control animals were euthanised by excess of inhaled isoflurane.

### 3.2. Brain Harvesting and Sectioning

Twenty-four hours or 48 h after the LPS treatment, the animals were anaesthetised by isoflurane inhalation in O_2_/NO_2_ and decapitated. The brains were quickly removed and snap-frozen in 2-methylbutane. Each brain was stored in a −80° freezer until sectioning.

Each brain was sectioned using a Leica CM3050 S Cryostat at −22 °C. Sections with a thickness of 12 µm were taken from approximately Bregma −1.54 mm to Bregma −1.76 mm, covering the inflammation region. The inflammation region was clearly observed during sectioning, near the coordinates where the LPS was injected. There was no inflammation observed in the control brains.

Coronal serial sections from each brain were thaw-mounted onto ITO-coated slides (Sigma Aldrich, Poole, UK) for MALDI and TOF-SIMS analyses. Serial sections were also thaw-mounted onto Super Frost Plus adhesion slides (Thermo Fisher Scientific, Winsford, UK) for DESI analysis and immunofluorescence staining.

For DESI imaging, the brain sections were taken out of the −80 °C freezer, thawed, and analysed immediately after. For TOF-SIMS experiments, the frozen sections were desiccated at room temperature for 15 min prior to analysis. For MALDI imaging, frozen sections were desiccated at room temperature for 15 min immediately prior to matrix application.

#### 3.2.1. Histological Staining

Brain sections were stained in 95% ethanol for 30 s, 70% ethanol for 30 s and deionised water for 30 s before Gill’s Haematoxylin staining (Sigma Aldrich, Poole, UK) for 3 min. After that, sections were rinsed for 3 min under running tap water, in 70% ethanol for 30 s and in 95% ethanol for 30 s. Counterstaining was applied by immersing the slides in Eosin Y (Sigma Aldrich, Poole, UK) for 1 min followed by 95% ethanol and 100% ethanol, 30 s each. Finally, the samples were cleared with xylene for 1 min and the slides were subsequently sealed with Organo/Limonene Mount (Sigma Aldrich, Poole, UK) and a cover slip.

The slides were scanned at the Bioimaging facility, The University of Manchester using a 3D-Histech Pannoramic-250 microscope slide-scanner with 40x magnification colour camera.

#### 3.2.2. Immunofluorescence

In this study, Iba1 was used as a microglia/macrophage marker. This protein is expressed in resting and activated microglia of the central nervous system [49]. To obtain microscopy images, a secondary antibody with a fluorescent fluorophore was used.

To stain the sections, the samples were fixed for 5 min in 4% PFA followed by 3 × 10 min in 10 mM PBS (phosphate-buffered saline). After that, the sections were permeabilised in 10 mM PBS with 0.1% Triton X-100 for 5 min. Non-specific binding was blocked with a protein blocking reagent (Animal-Free Blocker^®^ Concentrate, SP-5030-250, Vector Labs, California, USA) for 10 min. The slides were incubated with the primary antibody Iba1 (Wako 01919741, 1:300 in buffer) overnight at 4 °C. The following day, slides were washed 3 × 5 min in TBS (Tris-buffered saline) followed by incubation in the dark for 2 h with the secondary antibody Anti-Rabbit Alexa-647 (Invitrogen A21245, 1:500 in buffer). After that, the slides were incubated with 4′,6-diamidino-2-phenylindole (DAPI) for 5 min. The samples were rinsed for 3 × 5 min in TBS before mounting using Prolong gold antifade mounting medium (Thermo Fisher Scientific, Winsford, UK) and a coverslip. The sample slides were scanned using the Zeiss Axioscan (Zeiss Group, Jena, Germany) microscope slide scanner.

### 3.3. Mass Spectrometry Imaging (MSI)

#### 3.3.1. Desorption Electrospray Ionisation (DESI) Imaging and MS/MS

Brain sections were analysed with a Synapt G2-Si system (Waters Corporation, Milford, MA, USA), calibrated with Sodium formate in electrospray configuration. Untargeted images were acquired in sensitivity mode to enhance the detection of species as opposed to using resolution or high-resolution mode for acquisition. This resulted in higher signal with mass accuracy between 10 and 20 ppm, since the samples were large and the mass accuracy can vary during the analysis of samples with complex chemistry and morphology. For DESI imaging, a constant flow of solvent (5 µL/min, 98% methanol 2% water) and gas (Nitrogen at 6 bar) was used during the experiments. This flow was delivered by the DESI sprayer positioned at 75° of inclination, ~4 mm above the sample surface and with 0.7 kV in capillary. Imaging was carried out in both positive and negative ion mode, acquiring with a scan rate of 4 scans/second and spatial resolution set to 100 μm.

*DESI-MS/MS—*After performing DESI-MSI, selected ions were fragmented for mass assignment confirmation. The trap and the transfer collision energies were 35 eV and 2 eV for all the lipid species fragmented. The MS/MS window was adjusted to each precursor ion. The DESI spray and imaging conditions were the same as described above.

#### 3.3.2. Matrix-Assisted Laser Desorption Ionisation (MALDI) Imaging

Serial brain sections to those analysed with DESI-MSI were analysed with a MALDI-TOF RapifleX^®^ system (Bruker Corporation, Billerica, MA, USA) equipped with a Smartbeam 3D 355 nm Nd:YAG laser. Complete brain sections were imaged in positive ion mode with 35% laser power and 500 shots with a frequency of 10 kHz. The spatial resolution used was 50 µm and the mass range acquired was 120–1200 Da.

Matrix preparation- 2,5-Dihydroxybenzoic acid (Sigma Aldrich, Poole, UK) was prepared as a solution with 10 mg/mL in 70:30 methanol:0.1% trifluoroacetic acid in water (*v*/*v*).

Matrix application- The HTX M5 SprayerTM (HTX Technologies, LLC, Chapel Hill, NC, USA) located in the same lab was used to spray the MALDI matrix. 90 µL of the matrix solution were sprayed on each pass, with a velocity of 1200 and a temperature of 75 °C in nozzle. 12 passes were conducted per sample slide.

#### 3.3.3. Time-of-Flight Secondary Ion Mass Spectrometry (TOF-SIMS) Imaging

Higher-spatial resolution images were acquired on serial sections to those analysed with DESI and MALDI using a J105 Chemical Imager (Ionoptika Ltd., Southampton, UK). This instrument is equipped with a 70 kV gas cluster ion beam (Ionoptika Ltd., Southampton, UK) capable of providing a water cluster size of several thousands of molecules. For this study, clusters with 25,000 water molecules were used to achieve a beam with a diameter of 5 µm for imaging in negative ion mode.

### 3.4. Data Processing

MassLynx™ (Waters corporation, Massachusetts, USA) was used to process spectra obtained from DESI images. SCiLS Lab (SCiLS™ Lab, Bremen, Germany) was used to reconstruct images from all modalities and to generate single ion images, regions of interest, segmentation by bisecting k-means and image registration between MSI and microscopy images. The Ionoptika Analyser (Ionoptika Ltd., Southampton, UK) was used to generate single ion images from the data sets acquired with TOF-SIMS. All MSI data were normalised to the total ion count (TIC). Lipid species were assigned according to their accurate masses using the LIPID MAPS MS data bulk search tool [22].

H&E microscopy images were processed using SlideViewer (3DHISTECH Ltd., Budapest, Hungary). Immunofluorescence images were processed and exported using ZEISS Zen LITE (Zeiss Group, Jena, Germany)

## 4. Conclusions

- The application of multi-modal imaging techniques to study LPS-induced neuroinflammation models, such as MSI and microscopy, showed the up-regulation of bioactive lipid species and their accumulation in the inflamed hemisphere of the brain. These lipids could play a role during neuroinflammation as mediators of the immune response.
- The presence of NAPE lipids and microglia at the injury site could be related to the eCB signalling system. NAPE lipids, upregulated by microglia, might play a role as anti-inflammatory mediators inducing neuroprotection and reducing neuronal death.
- Accumulation of PUFAs and their derivatives and their previously described role as precursors of anti-inflammatory mediators indicate that these molecules are likely to modulate the neuroinflammatory response in the brain. This is, also further supported by their co-localisation with microglia observed with immunofluorescence.
- The accumulation of acylcarnitines close to the injury site suggests a deficiency in the carnitine transport and β-oxidation cycle. Acylcarnitines could also activate a pro-inflammatory response during neuroinflammation.
- The importance of detecting endogenous bioactive lipid species during the progression of neuroinflammation lies in the possibility of deconvoluting their role during the immune response to develop targeted therapeutics.

## Data Availability

The datasets used in the current study are available from the corresponding author upon request.

## Supplementary Materials

The following supporting information can be downloaded at: https://www.mdpi.com/article/10.3390/ijms252212032/s1.
