# Supplementary material for: Accumulation of Bioactive Lipid Species in LPS-Induced Neuroinflammation Models Analysed with Multi-Modal Mass Spectrometry Imaging
Source: Int J Mol Sci. 2024 Nov 8;25(22):12032. doi: 10.3390/ijms252212032 (PMC11594259; doi:10.3390/ijms252212032)
Supplement: Supplementary file 1 [file ijms-25-12032-s001.zip › ijms-3221702-supplementary.pdf]

Accumulation of bioactive lipid species in LPS-induced neuroinflammation models analysed with multi-modal mass spectrometry imaging

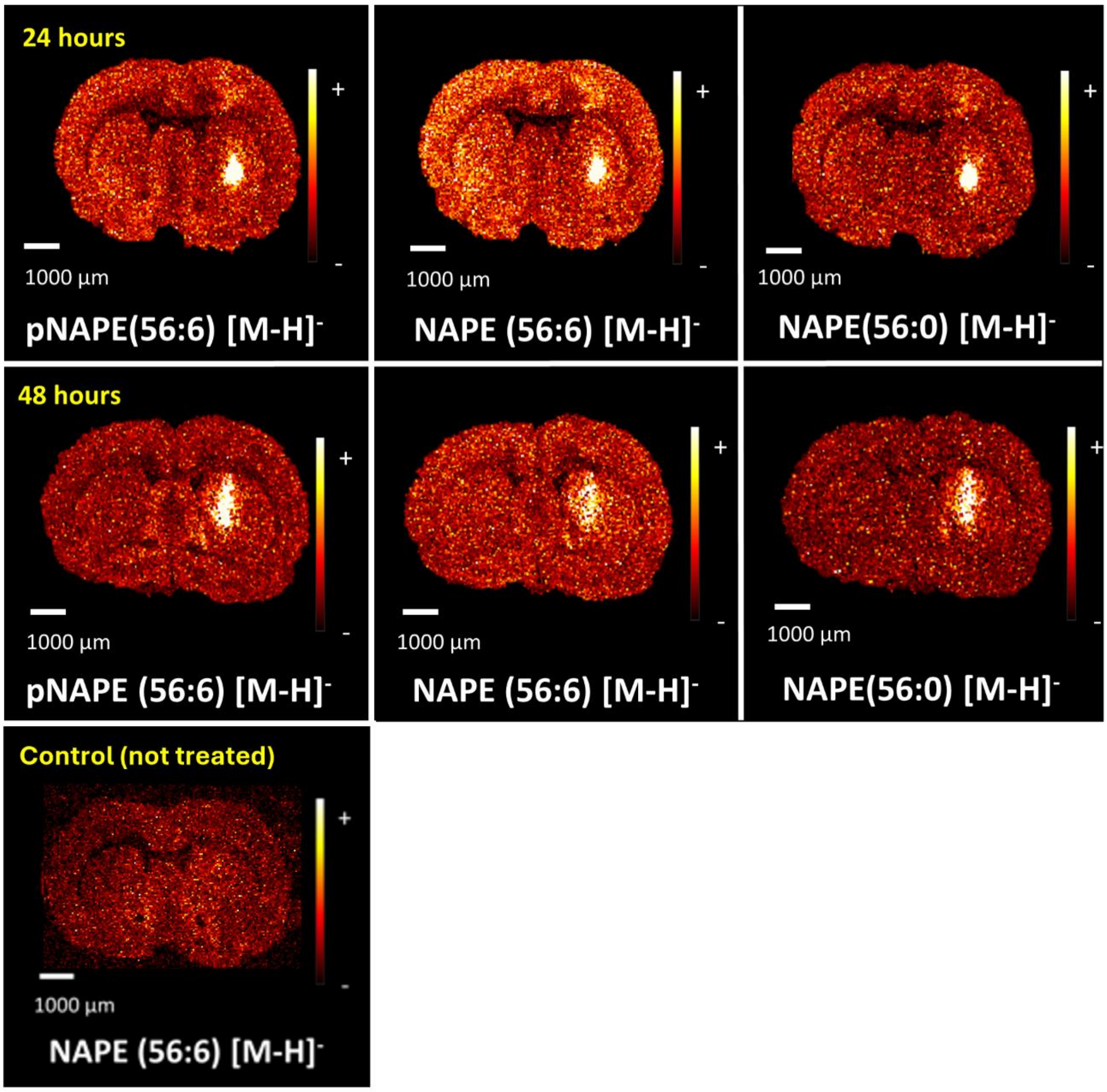

Figure S1: Single ion images obtained with DESI-MS. The segmentation maps were obtained with bisecting k-mean clustering to separate the region of inflammation (seen in blue/grey) from the rest of the brain structures (yellow/orange) and background substrate (red). Single ion images show the detection of N-Acylphosphatidylethanolamine (NAPE) lipid species with DESI Imaging in negative ion mode. pNAPE(56:6), NAPE(56:6) and NAPE(56:0) were detected mainly within the inflammation area after 24 hours and 48 hours of inducing the neuronflammatory event with LPS.

Putative assignment	Chemical formula [M]	Accurate m/z value [M-H]$^-$	Detected m/z [M-H]$^-$	Δ Mass (ppm)
NAPE(52:0)	$C_{57}H_{112}NO_9P$	984.7996	984.7728	27.21
NAPE(54:4)	$C_{59}H_{108}NO_9P$	1004.7686	1004.8004	31.64
pNAPE(56:6)*	$C_{61}H_{108}NO_8P$	1012.7734	1012.8035	29.72
NAPE (56:6)*	$C_{61}H_{108}NO_9P$	1028.7683	1028.7957	26.63
NAPE(56:4)	$C_{61}H_{112}NO_9P$	1032.7996	1032.8158	15.68
NAPE(56:0)*	$C_{61}H_{120}NO_9P$	1040.8622	1040.8512	10.56
NAPE(58:6)	$C_{63}H_{111}NO_9P$	1056.7996	1056.8457	43.62
*Annotation based on MS/MS analysis. The rest of the assignment was based on accurate masses.				

Table S1 List of NAPE lipid species detected as deprotonated [M-H]$^-$ molecules with DESI-MSI.

m/z	Putative assignment
960.779	N/A
984.7728	NAPE(52:0)
986.7772	N/A
988.8022	N/A
1000.7559	N/A
1004.8004	NAPE(54:4)
1010.7631	N/A
1012.8035	pNAPE(56:6)
1014.8142	N/A
1016.8459	N/A
1028.7957	NAPE (56:6)
1032.8158	NAPE(56:4)
1038.8235	N/A
1040.8512	NAPE(56:0)
1042.848	N/A
1044.8661	N/A
1056.8457	NAPE(58:6)

Table S2: m/z values co-localised to the inflammation regions with Person correlation analysis.

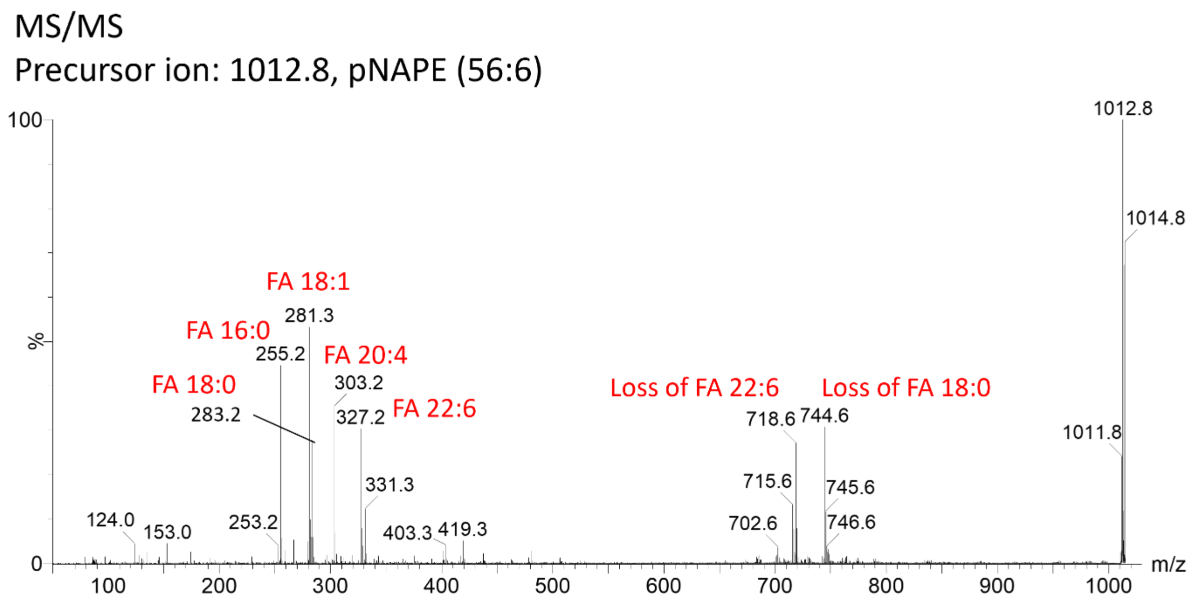

Figure S2: Combined MS/MS spectrum of pNAPE(56:6) acquired with DESI across the inflammation region of a brain section.

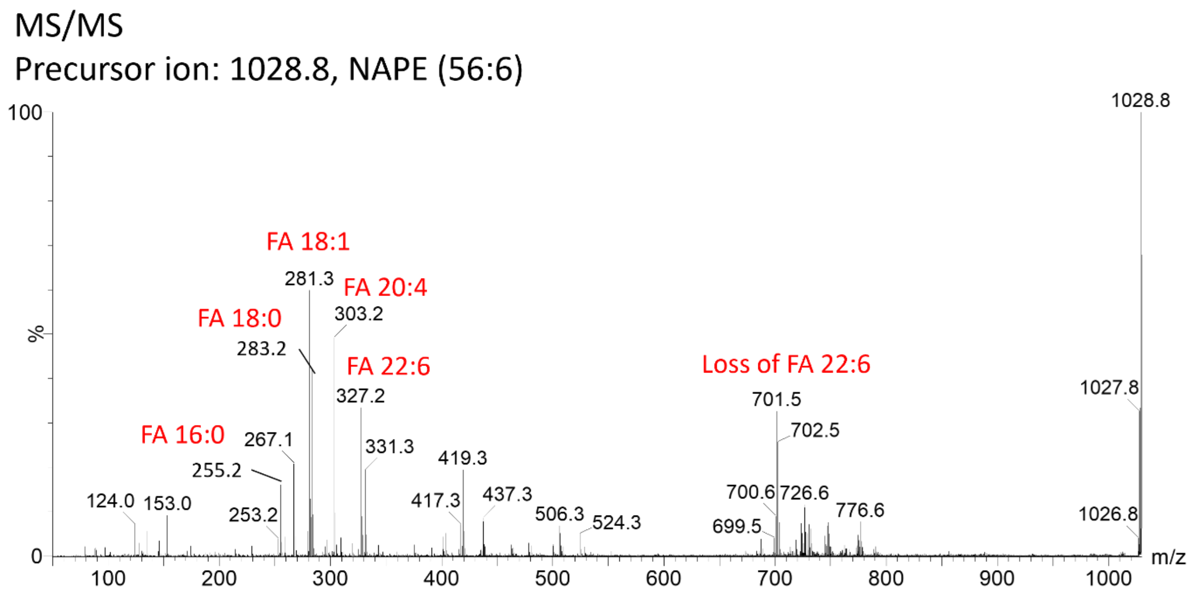

Figure S3: Combined MS/MS spectrum of NAPE(56:6) acquired with DESI across the inflammation region of a brain section.

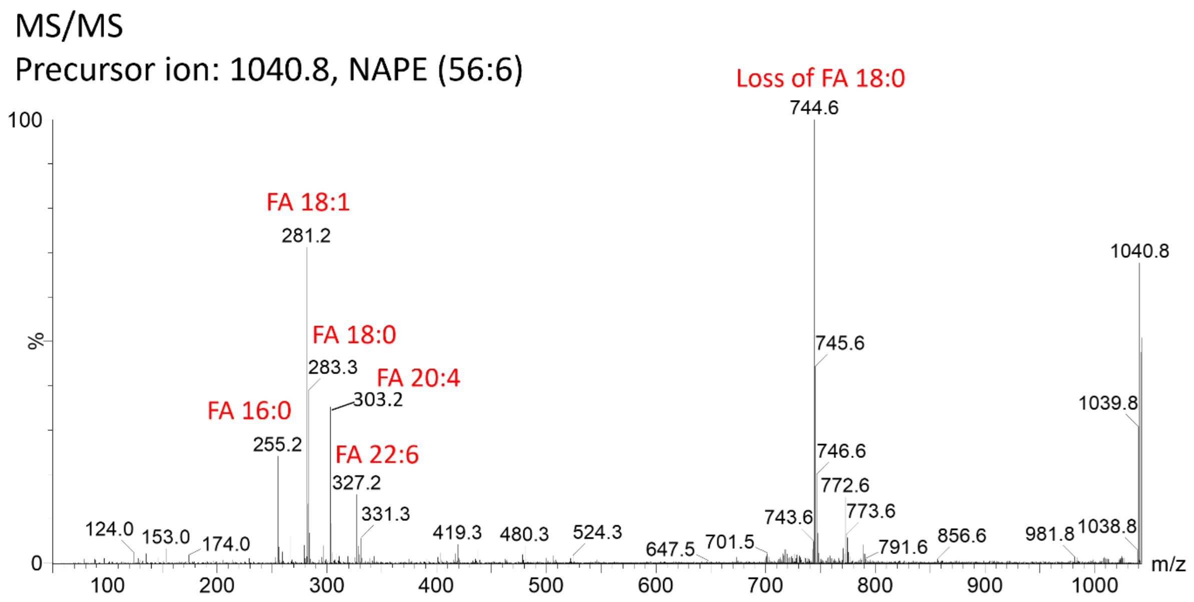

Figure S4: Combined MS/MS spectrum of NAPE(56:0) acquired with DESI across the inflammation region of a brain section.

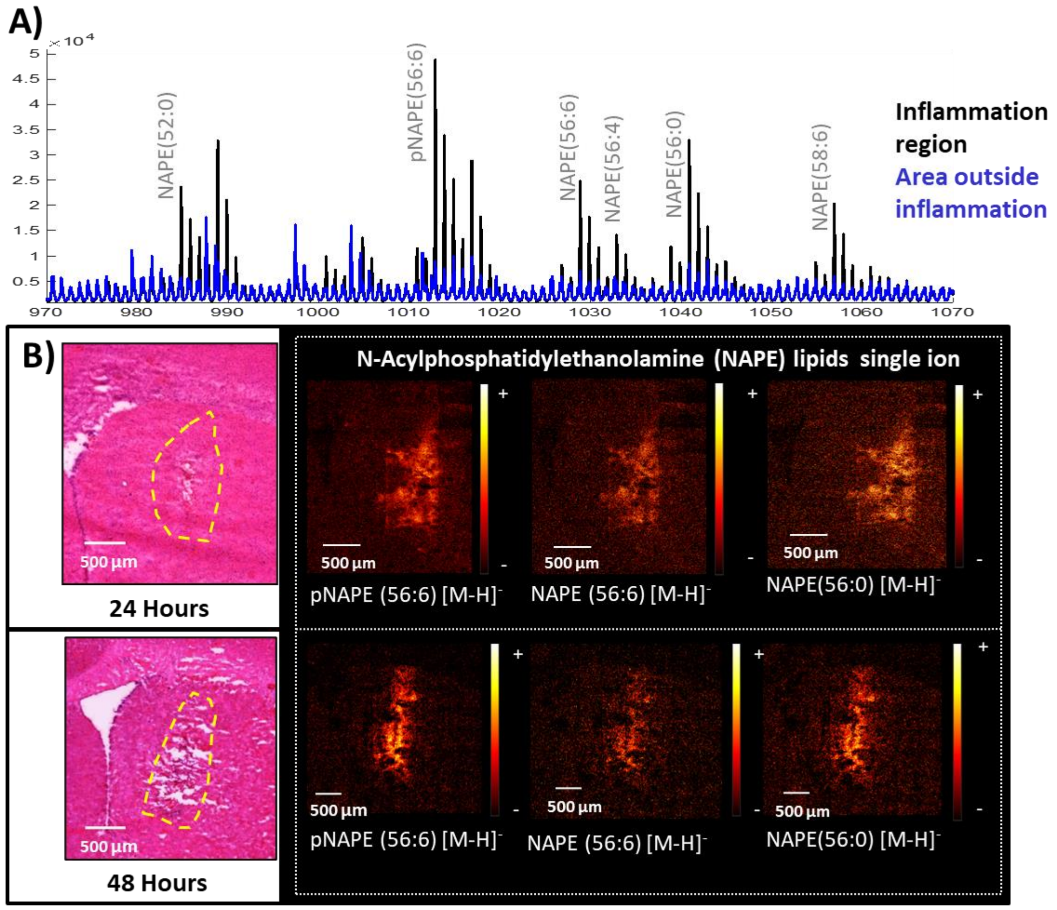

Figure S5: Overlaid Spectra acquired with TOF-SIMS, the black spectrum was extracted from the inflammation region whereas the blue spectrum was extracted from a region without inflammation. Both areas have the same number of pixels for comparison purposes. When comparing the spectra, the main chemical differences can be observed within the mass range m/z 970 to m/z 1100. Here, N-Acylphosphatidylethanolamine (NAPE) lipid species have been identified. B) Single ion images of selected NAPEs, accumulated mainly within the region of inflammation after 24 hours and 48 hours after LPS treatment. Microscopy images from consecutive sections from the same brains stained with H&E are also shown as a reference, with the inflammation region highlighted in yellow dotted areas.

PUFA species					
**Putative assignment**	**Chemical formula [M]**	**Accurate m/z value $[M-H]^-$**	**Detected m/z $[M-H]^-$**	**Δ Mass (ppm)**	**Lipid Maps ID**
DHA	$C_{22}H_{32}O_2$	327.2330	327.2362	9.78	LMFA01030185
DPA	$C_{22}H_{34}O_2$	329.2486	329.2494	2.43	LMFA04000044
EPA	$C_{20}H_{30}O_2$	301.2173	301.2225	17.26	LMFA01030759
Arachidonic acid	$C_{20}H_{32}O_2$	303.2330	303.2373	14.18	LMFA01030001
**Derivatives**					
**Putative assignment**	**Chemical formula [M]**	**Accurate m/z value $[M-H]^-$**	**Detected m/z $[M-H]^-$**	**Δ Mass (ppm)**	
Hydroxy-DHA	$C_{22}H_{32}O_3$	343.2279	343.2350	20.69	
Hydroxy-DPA	$C_{22}H_{34}O_3$	345.2430	345.2573	4.14	
Hydroxy-EPA	$C_{20}H_{30}O_3$	317.2117	317.2158	12.93	
Dihydroxy-DHA	$C_{22}H_{32}O_4$	359.2228	359.2248	5.57	
Dihydroxy-DPA	$C_{22}H_{34}O_4$	361.2384	361.2421	10.24	
Dihydroxy-EPA	$C_{20}H_{30}O_4$	333.2066	333.2130	19.21	
Dihydroxy-AA	$C_{20}H_{32}O_4$	335.2222	335.2295	21.78	

Table S3: Assignment of PUFA species and their derivatives

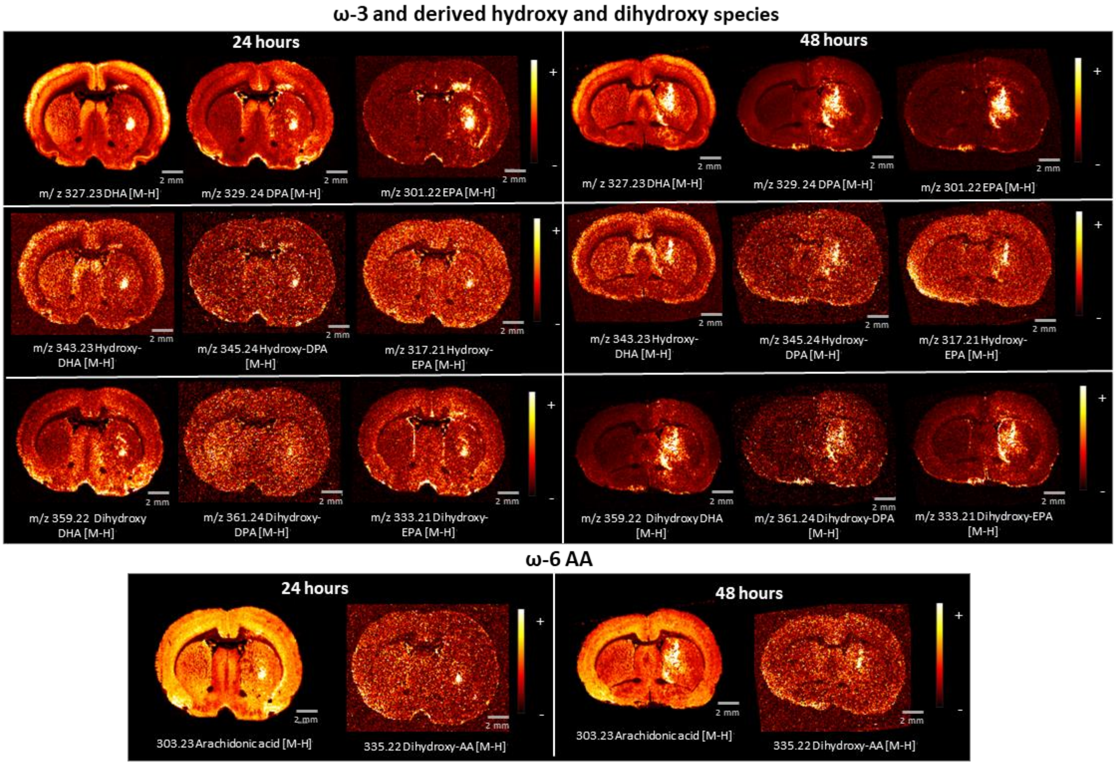

Figure S6: Accumulation of PUFAs and their hydroxy and Dihydroxy derivatives in brains injected with LPS.

Putative assignment	Chemical formula [M]	Accurate m/z value [M+H]+	Detected m/z [M+H]+	Δ Mass (ppm)	Lipid Maps ID
CAR(14:0)	$C_{21}H_{41}NO_4$	372.3108	372.3132	6.44	LMFA07070102
CAR(16:0)	$C_{23}H_{45}NO_4$	400.3421	400.3453	7.99	LMFA07070098
CAR(18:1)	$C_{25}H_{47}NO_4$	426.3578	426.3614	8.44	LMFA07070096<br>LMFA07070063
CAR(18:0)	$C_{25}H_{49}NO_4$	428.3734	428.3719	3.50	LMFA07070051

Table S4: Assignment of Acylcarnitine species and their derivatives

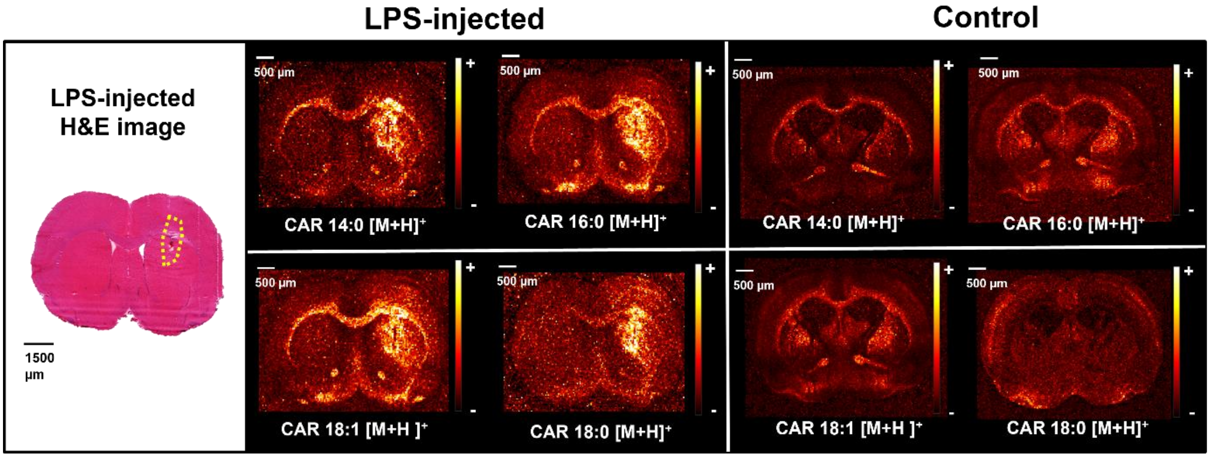

Figure S7: Accumulation of Fatty acyl carnitines surrounding the inflammation region detected with DESI-MS. (Right) H&E microscopy image showing the inflammation region. (Left) Single ion images showing the detection of tetradecanoylcarnitine (CAR 14:0) at m/z 372.31, palmitoylcarnitine (CAR 16:0) at m/z 400.34, elaidic carnitine or O-oleoylcarnitine (CAR 18:1) at m/z 426.35 and stearoylcarnitine (CAR 18:0) at m/z 428.37.
